# Genome sequences of 71 ecologically and geographically diverse *Enterobacter* strains

**DOI:** 10.1128/mra.00334-26

**Published:** 2026-07-10

**Authors:** Sara Jordan, Joël F. Pothier, Pieter de Maayer, Brian H. Kvitko, Teresa A. Coutinho, Theo H. M. Smits

**Affiliations:** 1 Environmental Genomics and Systems Biology Research Group, Institute for Environment and Natural Resources, Zurich University for Applied Sciences (ZHAW), Wädenswil, Switzerland; 2 School of Molecular and Cell Biology, University of the Witwatersrand, Johannesburg, South Africa; 3 Department of Plant Pathology, University of Georgia 1355, , Athens, Georgia, USA; 4 Department of Microbiology and Plant Pathology, Centre for Microbial Ecology and Genomics/Forestry and Agricultural Biotechnology Institute (FABI), University of Pretoria 56410, Pretoria, South Africa; Fluxus Inc., Sunnyvale, California, USA

**Keywords:** Enterobacteriaceae, onion isolates, clinical isolates, food isolates

## Abstract

We sequenced the genomes of 77 bacterial strains, tentatively identified as Enterobacter. Of these, 71 were identified as *Enterobacter* based on genomic analysis. These strains span 11 species of diverse ecological and geographical origin.

## ANNOUNCEMENT

*Enterobacter* spp. are Gram-negative bacteria found in a wide range of environments and are best known as opportunistic human pathogens (1). Aside from their clinical relevance, some members are also recognized as plant pathogens, plant growth promoters, or biocontrol agents (2). Although the genomes of many clinical strains have been sequenced, genomes of plant-associated strains remain underrepresented. The strains sequenced in this study were isolated from a wide range of sources between 1929 and 2024, many of them originating from diseased onions (3).

Genomic DNA was extracted from cells grown overnight at 28°C in Luria-Bertani medium using the Gentra PureGene Yeast/Bact kit protocol (Qiagen, Hilden, Germany). The genomic DNA was quantified using the DeNovix dsDNA High Sensitivity Fluorescent Assay Kit (DeNovix, Wilmington, DE) and a Fluo-100B fluorometer (Allsheng). For short-read sequencing, library preparation was carried out with the Nextera XT DNA library prep kit (Illumina, San Diego, USA) following the manufacturer’s instructions. Sequencing was performed on a MiSeq sequencer (paired-end; 300 bp) using MiSeq v3 reagent (Illumina, San Diego, USA). The resulting paired-end reads were quality-controlled using FastQC version 0.11.9. Library preparation for long-read sequencing (ONT) was done with the ligation sequencing kit V14 (Oxford Nanopore Technologies, Oxford, United Kingdom) without shearing and size-selection. The native barcoding kit 24 V14 was used for multiplexing, and the sequencing was performed on a MinION Mk1B sequencer with an R10.4.1 flow cell. Base calling was performed using Guppy version 6.4.6, the high accuracy model v3.5.2-400bps. For two of the strains, ONT sequencing was outsourced to Microsynth AG (Table 1).

**TABLE 1:** Strain information and metrics of the sequenced genomes

Strain	Species	Isolation source	Geographical location	Year	Genome status	Number of contigs	Genome size	N_50_	Genome coverage (×)	GC (%)	BUSCO scores (%)^*a*^	Assembly accession	N predicted genes	Sequencing technology	Number of reads	ONT N_50_	SRA accession
20CA0162	*Enterobacter asburiae*	Onion leaf	USA: California	2020	Complete	1	4,537,160	4,537,160	177	56.07	99.1	CP196789	4,349	MiSeq	984588		SRR34071722
														MinION	57897	10455	SRR34071659
20WA0044	*Enterobacter asburiae*	Onion neck	USA: Washington State	2020	Complete	2	4,599,182	4,544,143	206	55.95	99.1	CM136142	4,424	MiSeq	882965		SRR34071719
														MinION	174976	3622	SRR34071656
VA26710	*Enterobacter asburiae*	superficial wound (foot)	Switzerland: Bellinzona	2011	Complete	1	4,565,988	4,565,988	110	56.09	99.1	CP197273	4,388	MiSeq	887141		SRR34071711
														MinION	22833	10676	SRR34071684
20T X 0138	*Enterobacter bugandensis*	Onion bulb	USA: Texas	2020	Complete	2	4,750,444	4,745,481	311	55.98	99.1	CP196779–CP196780	4,526	MiSeq	1368369		SRR34071729
														MinION	180887	4454	SRR34071665
20T X 0143	*Enterobacter bugandensis*	Onion bulb	USA: Texas	2020	Complete	2	4,860,553	4,795,896	120	55.88	99.1	JBSBBU000000000	4,702	MiSeq	749656		SRR34071727
														MinION	54634	6914	SRR34071663
U26840	*Enterobacter bugandensis*	urine	Switzerland: Bellinzona	2011	Complete	2	4,849,363	4,721,271	113	56.16	99.1	CP196773–CP196774	4,636	MiSeq	975614		SRR34071712
														MinION	20716	12908	SRR34071649
21PA0076	*Enterobacter cancerogenus*	Onion bulb	USA: Pennsylvania	2021	Draft	32	4,893,013	500,071	158	55.71	99.1	JBPGLG000000000	4,708	MiSeq	1638858		SRR34071705
S6	*Enterobacter cloacae*	Onion bulb	Gambia	2024	Draft	48	4,840,494	265,512	90	54.98	99.1	JBPGKG000000000	4,626	MiSeq	1031991		SRR34071672
SU1	*Enterobacter cloacae*	Onion bulb	South Africa: Pretoria	2024	Draft	46	4,907,882	281,553	130	54.86	99.1	JBPGKI000000000	4,658	MiSeq	1888942		SRR34071674
SU8	*Enterobacter cloacae*	Onion bulb	South Africa: Pretoria	2024	Draft	31	4,905,795	516,868	71	55.08	99.1	JBPGKH000000000	4,672	MiSeq	800292		SRR34071673
21T X 0121	*Enterobacter cloacae* subsp. *cloacae*	Onion leaf	USA: Texas	2021	Draft	46	4,791,301	292,095	133	55.11	99.1	JBPGLE000000000	4,540	MiSeq	1189006		SRR34071702
NRRL B-41756	*Enterobacter hormaechei* subsp. *hoffmannii*	ground beef	USA: Lewistown	2006	Draft	81	4,909,095	181,187	114	55.11	99.1	JBPGKV000000000	4,799	MiSeq	1030934		SRR34071693
20T X 0131	*Enterobacter hormaechei* subsp. *steigerwaltii*	Onion bulb	USA: Texas	2020	Draft	26	4,719,003	468,456	77	55.53	98.8	JBPGLV000000000	4,514	MiSeq	634912		SRR34071744
20T X 0139	*Enterobacter hormaechei* subsp. *steigerwaltii*	Onion bulb	USA: Texas	2020	Complete	2	4,760,511	4,624,588	261	55.52	98.8	CP196777–CP196778	4,541	MiSeq	1096151		SRR34071728
														MinION	151514	4937	SRR34071664
NRRL B-51002	*Enterobacter hormaechei subsp. steigerwaltii*	ground beef	USA: Peoria	2007	Draft	43	4,701,572	338,538	74	55.67	98.8	JBPGKT000000000	4,533	MiSeq	65336		SRR34071690
NRRL B-59360	*Enterobacter hormaechei* subsp. *steigerwaltii*	ground beef	USA: Peoria	2009	Draft	74	4,900,220	264,91	62	55.37	98.8	JBPGKM000000000	4,823	MiSeq	720838		SRR34071679
VU2	*Enterobacter hormaechei* subsp. *steigerwaltii*	Onion bulb	South Africa: Pretoria	2024	Draft	66	4,795,089	216,681	51	55.45	98.8	JBPGKK000000000	4,650	MiSeq	603729		SRR34071676
21T X 0095	*Enterobacter hormaechei* subsp. *xiangfangensis*	Onion bulb	USA: Texas	2021	Draft	104	4,544,083	114,589	24	55.52	98.8	JBPGKP000000000	4,364	MiSeq	269509		SRR34071686
A10a	*Enterobacter kobei*	Onion bulb	USA: New York	2010	Draft	23	4,776,380	529,008	39	55.04	99.1	JBPGMH000000000	4,614	MiSeq	319184		SRR34071748
AZ3a	*Enterobacter kobei*	Onion bulb	USA: New York	2015	Complete	2	4,870,976	4,716,038	221	55.05	99.1	CP196783–CP196784	4,680	MiSeq	962976		SRR34071732
														MinION	91204	11061	SRR34071669
B03a	*Enterobacter kobei*	Onion bulb	USA: New York	2016	Complete	2	4,815,610	4,694,372	120	55.04	99.1	CP196781–CP196782	4,606	MiSeq	687299		SRR34071731
														MinION	36549	14305	SRR34071668
BA1c	*Enterobacter kobei*	Onion bulb	USA: New York	2015	Draft	28	4,634,850	344,465	60	55.12	99.1	JBPGMD000000000	4,443	MiSeq	552656		SRR34071714
NRRL B-41482	*Enterobacter kobei*	chive leaf	USA: Washington	2006	Draft	160	4,952,117	105,766	121	54.81	99.3	JBPGKW000000000	4,932	MiSeq	1087240		SRR34071694
U27616	*Enterobacter kobei*	urine	Switzerland: Bellinzona	2011	Complete	1	4,769,390	4,769,390	119	54.90	99.3	CP196786	4,547	MiSeq	917404		SRR34071713
														MinION	31853	10327	SRR34071650
19UT002	*Enterobacter ludwigii*	Onion bulb	USA: Utah	2019	Complete	1	4,877,601	4,877,601	99	54.59	99.3	CP196787	4,673	MiSeq	759803		SRR34071715
														MinION	27975	10044	SRR34071651
20CA0144	*Enterobacter ludwigii*	Onion bulb	USA: California	2020	Draft	30	4,843,254	513,725	80	54.52	99.1	JBPGLS000000000	4,696	MiSeq	669115		SRR34071741
20CA0165	*Enterobacter ludwigii*	Onion leaf	USA: California	2020	Complete	6	5,266,515	4,779,747	145	54.22	99.4	JBPGLL000000000	5,064	MiSeq	1326716		SRR34071721
														MinION	33620	12812	SRR34071658
20CA0169	*Enterobacter ludwigii*	Onion neck	USA: California	2020	Draft	25	4,752,938	518,867	133	54.72	99.1	JBPGLO000000000	4,568	MiSeq	1210107		SRR34071735
20CA0178	*Enterobacter ludwigii*	Onion bulb	USA: California	2020	Draft	20	4,812,377	788,539	72	54.70	99.1	JBPGLR000000000	4,648	MiSeq	80305		SRR34071740
20GA0256	*Enterobacter ludwigii*	Onion leaf	USA: Georgia	2020	Draft	73	5,615,739	305,875	113	53.75	99.4	JBPGLN000000000	5,540	MiSeq	1170001		SRR34071734
20T X 0154	*Enterobacter ludwigii*	Onion bulb	USA: Texas	2020	Draft	34	4,927,652	608,923	74	54.49	99.3	JBPGLU000000000	4,780	MiSeq	665396		SRR34071743
20T X 0174	*Enterobacter ludwigii*	Onion bulb	USA: Texas	2020	Complete	2	4,799,291	4,687,065	144	54.64	99.1	CP196775–CP196776	4,638	MiSeq	843303		SRR34071726
														MinION	45758	11844	SRR34071662
20T X 0177	*Enterobacter ludwigii*	Onion bulb	USA: Texas	2020	Draft	28	4,758,082	660,857	685	54.65	99.1	JBPGLT000000000	4,604	MiSeq	5758437		SRR34071742
20WA0021	*Enterobacter ludwigii*	Onion neck	USA: Washington State	2020	Draft	48	4,814,481	354,125	50	54.55	98.8	JBPGLM000000000	4,692	MiSeq	57178		SRR34071733
20WA0163	*Enterobacter ludwigii*	Onion bulb	USA: Washington State	2020	Draft	19	4,884,018	2,669,397	101	54.57	99.1	JBPGLQ000000000	4,754	MiSeq	901593		SRR34071738
20WA0227	*Enterobacter ludwigii*	Onion neck	USA: Washington State	2020	Draft	30	4,946,116	491,368	197	54.52	99.3	JBPGLP000000000	4,780	MiSeq	1766731		SRR34071737
21NY0087	*Enterobacter ludwigii*	Onion bulb	USA: New York	2021	Draft	38	4,966,246	388,099	128	54.55	99.1	JBPGLJ000000000	4,805	MiSeq	1414748		SRR34071708
AM27	*Enterobacter ludwigii*	Stored water	South Africa: Limpopo	2021	Draft	29	5,005,215	748,426	114	54.5	99.1	JBPGMF000000000	4,883	MiSeq	1065378		SRR34071736
CG66	*Enterobacter ludwigii*	Onion transplants	USA: Georgia	2018	Draft	25	4,758,674	637,787	99	54.61	99.3	JBPGME000000000	4,574	MiSeq	865036		SRR34071725
I59	*Enterobacter ludwigii*	Onion bulb	USA: New York	2011	Draft	36	4,921,544	525,102	136	54.55	99.3	JBPGMA000000000	4,761	MiSeq	1256590		SRR34071677
LE1	*Enterobacter ludwigii*	retail leek	Switzerland	2024	Complete	1	4,736,143	4,736,143	125	54.68	99.1	JBSBBW000000000	4,563	MiSeq	133038		SRR34071671
														PromethION^*b*^	80915	11999	SRR34071682
M79a	*Enterobacter ludwigii*	Onion bulb	USA: New York	2011	Draft	84	5,592,069	424,349	148	53.93	99.3	JBPGMB000000000	5,414	MiSeq	1503571		SRR34071692
NRRL B-115	*Enterobacter ludwigii*	Unknown	Unknown	1929	Draft	26	4,679,292	445,036	52	54.70	99.1	JBPGKO000000000	4,503	MiSeq	577111		SRR3407168
NRRL B-226	*Enterobacter ludwigii*	Unknown	Unknown	1941	Draft	46	4,751,936	412,196	151	54.73	99.1	JBPGLD000000000	4,607	MiSeq	80915		SRR34071701
NRRL B-23266	*Enterobacter ludwigii*	compost	USA: Peoria	1998	Draft	34	4,705,490	487,621	132	54.71	99.1	JBPGLA000000000	4,493	MiSeq	1152883		SRR34071698
NRRL B-23289	*Enterobacter ludwigii*	garden soil	USA: Peoria	1998	Draft	26	4,930,699	489,916	129	54.47	99.1	JBPGKZ000000000	4,743	MiSeq	1195874		SRR34071697
NRRL B-2744	*Enterobacter ludwigii*	Unknown	Unknown	1961	Draft	30	4,739,904	527,046	128	54.68	99.1	JBPGLB000000000	4,548	MiSeq	1082867		SRR34071699
NRRL B-322	*Enterobacter ludwigii*	Unknown	Unknown	1942	Draft	35	4,873,665	398,657	136	54.59	99.1	JBPGKN000000000	4,702	MiSeq	1621233		SRR34071680
NRRL B-413	*Enterobacter ludwigii*	water supply	USA: California	1942	Draft	31	4,636,424	323,895	111	54.81	99.1	JBPGLC000000000	4,459	MiSeq	897849		SRR34071700
NRRL B-51109	*Enterobacter ludwigii*	cod fish (retail, fresh)	USA: Peoria	2007	Draft	39	4,969,678	466,954	128	54.47	99.4	JBPGKS000000000	4,789	MiSeq	1206040		SRR34071689
NRRL B-59403	*Enterobacter ludwigii*	Unknown	USA: Peoria	2009	Draft	24	4,848,648	701,458	123	54.56	99.1	JBPGKY000000000	4,681	MiSeq	1127969		SRR34071696
NRRL B-59494	*Enterobacter ludwigii*	zea mays kernels	USA: Kilborne	2010	Draft	50	4,905,281	407,806	125	54.58	99.3	JBPGKX000000000	4,765	MiSeq	1151315		SRR34071695
NRRL B-59708	*Enterobacter ludwigii*	Zea may kernel	USA: Kilborne	2012	Draft	28	4,768,427	433,581	116	54.58	99.3	JBPGKR000000000	4,593	MiSeq	1109542		SRR34071688
ON18	*Enterobacter ludwigii*	retail onion	Switzerland	2024	Complete	1	4,732,544	4,732,544	14	54.60	96.6	JBSBBV000000000	4,542	PromethION^b^	11707	13915	SRR34071681
R54	*Enterobacter ludwigii*	Onion bulb	USA: New York	2011	Draft	35	4,875,122	570,272	286	54.36	99.3	JBPGMC000000000	4,733	MiSeq	2503417		SRR34071703
T16c	*Enterobacter ludwigii*	Onion bulb	USA: New York	2011	Draft	66	4,984,869	209,709	34	54.45	99.3	JBPGLX000000000	4,845	MiSeq	305555		SRR34071746
VA26405-12	*Enterobacter ludwigii*	gall puncture	Switzerland: Bellinzona	2011	Complete	2	4,847,272	4,761,571	246	54.75	99.1	CP196820–CP196821	4,628	MiSeq	1812172		SRR34071710
														MinION	151789	3632	SRR34071683
VA26405-13	*Enterobacter ludwigii*	gall puncture	Switzerland: Bellinzona	2011	Draft	51	4,798,334	489,097	441	54.72	99.1	JBPGLK000000000	4,615	MiSeq	5338744		SRR34071709
19UT013	*Enterobacter mori*	Onion bulb	USA: Utah	2019	Draft	48	4,807,575	251,87	149	55.45	98.8	JBPGLW000000000	4,550	MiSeq	1349013		SRR34071745
20CA0205	*Enterobacter mori*	Onion bulb	USA: California	2020	Complete	3	5,691,611	5,354,134	130	54.75	98.8	CP197270–CP197272	5,396	MiSeq	1092436		SRR34071720
														MinION	26669	11969	SRR34071657
20WA0184	*Enterobacter mori*	Onion neck	USA: Washington State	2020	Complete	1	5,128,947	5,128,947	262	55.10	98.8	JBSBBX000000000	4,883	MiSeq	2359313		SRR34071717
														MinION	84355	7971	SRR34071653
20WA0277	*Enterobacter mori*	Onion bulb	USA: Washington State	2020	Complete	1	5,192,571	5,192,571	171	55.09	99.1	CP197274	4,966	MiSeq	1793267		SRR34071716
														MinION	28544	9313	SRR34071652
SM29	*Enterobacter mori*	Stool	South Africa: Limpopo	2021	Draft	31	4,761,412	477,607	87	55.60	99.1	JBPGLY000000000	4,520	MiSeq	755072		SRR34071655
SM41	*Enterobacter mori*	Stool	South Africa: Limpopo	2021	Complete	1	4,806,858	4,806,858	313	55.58	99.1	CP197276	4,546	MiSeq	948554		SRR34071730
														MinION	189482	6972	SRR34071667
NRRL B-41899	*Enterobacter pasteurii*	lettuce leaf	USA: Peoria	2007	Draft	55	4,891,684	368,089	110	56.35	98.8	JBPGKU000000000	4,660	MiSeq	993269		SRR34071691
20CA0094	*Enterobacter roggenkampii*	Onion bulb	USA: California	2020	Complete	3	4,753,588	4,743,844	123	55.97	99.1	JBSBBT000000000	4,564	MiSeq	823679		SRR34071724
														MinION	43117	7772	SRR34071661
20CA0095	*Enterobacter roggenkampii*	Onion bulb	USA: California	2020	Complete	1	4,743,852	4,743,852	389	55.98	99.1	CP197275	4,547	MiSeq	1081206		SRR34071723
														MinION	187805	16234	SRR34071660
20WA0107	*Enterobacter roggenkampii*	Onion neck	USA: Washington State	2020	Complete	2	4,732,981	4,647,609	154	55.87	99.1	CP196818–CP196819	4,540	MiSeq	1296257		SRR34071718
														MinION	29639	12209	SRR34071654
VU4	*Enterobacter roggenkampii*	Onion bulb	South Africa: Pretoria	2024	Draft	70	4,768,022	189,876	56	55.98	99.1	JBPGKJ000000000	4,657	MiSeq	68002		SRR34071675
20GA0343	*Enterobacter wuhouensis*	Onion bulb	USA: Georgia	2020	Complete	1	4,900,714	4,900,714	141	56.24	98.9	CP196792	4,651	MiSeq	522804		SRR34071739
														MinION	49090	14554	SRR34071670
NRRL B-59707	*Enterobacter wuhouensis*	Zea may kernel	USA: Kilborne	2012	Draft	30	4,792,965	567,172	88	56.10	98.8	JBPGKL000000000	4,583	MiSeq	1037300		SRR34071678
SM06	*Escherichia coli*	Stool	South Africa: Limpopo	2021	Draft	116	4,677,455	118,479	158	50.8	99.8	JBPGLZ000000000	4,556	MiSeq	1378054		SRR34071666
21OR0018	*Kosakonia cowanii*	Onion leaf	USA: Oregon	2021	Draft	33	4,963,607	440,46	124	55.82	99.1	JBPGKQ000000000	4,762	MiSeq	1685631		SRR34071687
21OR0021	*Kosakonia cowanii*	Onion bulb	USA: Oregon	2021	Draft	36	4,971,076	365,529	136	55.80	99.1	JBPGLI000000000	4,773	MiSeq	1435301		SRR34071707
21OR0077	*Kosakonia cowanii*	Onion leaf	USA: Oregon	2021	Draft	32	4,865,133	662,676	111	56.12	99.1	JBPGLH000000000	4,593	MiSeq	971831		SRR34071706
21T X 0090	*Leclercia pneumoniae*	Onion bulb	USA: Texas	2021	Draft	22	4,433,398	515,21	154	55.01	99.3	JBPGLF000000000	4,225	MiSeq	1533099		SRR34071704
AM23	*Psychrobacter faecalis*	Stored water	South Africa: Limpopo	2021	Draft	154	3,142,528	55,866	172	43.46	98.5	JBPGMG000000000	2,733	MiSeq	1005956		SRR34071747

^*a*^ BUSCO, Benchmarking Universal Single-Copy Orthologs.

^*b*^ ONT sequencing by Microsynth (Library preparation with Rapid Barcoding Kit 96 (SQK-RBK114.96) and sequenced on a PromethION apparatus using a FLO-PRO114M flow cell).

Hybrid assemblies and short- or long-read-only assemblies were conducted with Unicycler version 0.4.9 (4). For hybrid assemblies in which a complete genome was not achieved, reads were assembled again with Trycycler version 0.5.3 (5) or Autocycler version 0.4.0 (6), then polished with Pilon version 1.24 (7). Determination of contig circularization and rotation of circular genomes was done by the assembler software. For Unicycler assemblies the circularization was confirmed with Platon version 1.7 (8). Assembly summary statistics were generated with QUAST version 5.2.0 (9) and completeness of the genomes was estimated using BUSCO version 5.4.3 (10) and the enterobacterales_odb10 or pseudomonadales_odb10 lineage. The genomes were annotated with the NCBI Prokaryotic Genome Annotation Pipeline (PGAP) version 6.10 (11). Species identity was determined by calculating average nucleotide identity against all *Enterobacter* type strain genomes using fastANI version 1.33 (12). For six strains that were not identified as *Enterobacter* spp. using this approach, the genome was submitted to the Type Strain Genome Server (13) for identification. All tools were run with default parameters.

Complete genomes with zero to five plasmids were generated for all 23 hybrid assemblies. Short-read-only assemblies resulted in draft genomes with 19–160 contigs. The long-read-only assembly had the lowest BUSCO completeness at 96.6%; however, it also has a very low genome coverage. For the other genomes, the BUSCO completeness was between 98.5% and 99.8%. The sizes of the *Enterobacter* genomes ranged from 4,537,160 to 5,691,611 bp, with GC between 53.75% and 56.35%. The sequenced genomes described here will be used for further comparative genomic analyses.

## Data Availability

The genome sequences have been deposited in NCBI under BioProject no. PRJNA1279084. The genome and raw read accession numbers for each isolate are shown in Table 1.
